# Time to Analgesia Provision for Abdominal Pain Presentations in the Emergency Department: The Effect of Biological Sex—A Retrospective Cohort Study

**DOI:** 10.1111/1742-6723.70249

**Published:** 2026-03-31

**Authors:** Lisa Walker, Hannah Pennicott, Chris J. Selman, Elyssia M. Bourke

**Affiliations:** ^1^ Emergency Department Royal Melbourne Hospital Parkville Victoria Australia; ^2^ Department of Critical Care University of Melbourne Parkville Victoria Australia; ^3^ Clinical Epidemiology and Biostatistics Unit Murdoch Children's Research Institute Melbourne Victoria Australia; ^4^ Emergency Department Grampians Health Ballarat Victoria Australia

**Keywords:** emergency medicine, gender, inequity, pain management

## Abstract

**Objective:**

Abdominal pain is a common emergency department (ED) presentation. Currently, there is limited Australian literature detailing whether biological sex results in differences in analgesia provision for abdominal pain. The primary aim of this study is to determine whether there is a difference in time to analgesia administration based on biological sex for patients presenting with abdominal pain to the ED.

**Methods:**

This was a retrospective, single centre cohort study of adult patients presenting to the Royal Melbourne Hospital ED between April 1st and 30th 2024 with abdominal pain. Data relating to the patient's presentation and management were collected from records of eligible patients.

**Results:**

Of 708 eligible patients, 292 (41%) were biologically male and 416 (59%) female. There were 559 (80%) patients who received at least one dose of analgesia. Females waited a median of 75 min and males 59 min to receive their first dose of analgesia (difference in medians = 16 min, 95% confidence interval [CI] 0.9–31.0 min, *p* = 0.04). Females were also nearly half as likely to receive parenteral analgesia (OR 0.56, 95% CI 0.38–0.82, *p* = 0.003). We found minimal differences in diagnoses, triage category or pain scores between sexes.

**Conclusion:**

Females presenting to the ED with abdominal pain are waiting longer than males to receive their first dose of analgesia and are less likely to receive parenteral analgesia. Further research is required to determine the reason for this discrepancy and to then remediate it.

## 1 Introduction

Abdominal pain is one of the most common reasons for patients to present to the emergency department (ED) [1]. Providing adequate and timely analgesia is essential for good quality patient care. The Australian National Health and Medical Research Council (NHMRC) recommend that the median time to analgesia following presentation to the ED is 30 min [2]. Previous research has demonstrated that there is often a delay to patients receiving analgesia and that the factors contributing to this are complex but may include age, triage category and biological sex [3, 4, 5]. Previous research has also identified a difference in the rate of opiate prescribing between the biological sexes, with males more likely to receive opiates [6, 7].

There have been few studies previously examining whether there is a difference in time to analgesia provision for patients presenting to the ED with abdominal pain based on the patient's biological sex. The studies that have been conducted suggest differences in the timing and amount of analgesia given between the biological sexes. However, these studies have been undertaken outside of Australia where there are different structural and provider practices [4] or in private EDs where the patient cohort and resourcing may differ from a public ED service [8].

In the current study we evaluate a retrospective cohort of patients presenting with abdominal pain to the Royal Melbourne Hospital ED, a public tertiary service, to assess the use of analgesia in this setting. Improving our understanding of when and how pain is treated pharmacologically in the ED for this cohort of patients is essential due to the commonality of this presentation and the focus on analgesia as a marker of high‐quality patient care.

Our study aims to describe whether there is a biological sex disparity in the administration of analgesia for patients presenting with abdominal pain. Specifically, the primary objective of this study is to determine whether there is a difference in time to initial analgesia based on biological sex. Secondary objectives include assessing for differences in assigned triage category and assigned numerical pain score between biological sex groups. We will also compare differences in analgesia administration route (oral, parenteral), type of analgesia given (no analgesia, non‐opioid, opioid), and differences in disposition.

## 2 Methods

### 2.1 Study Design and Setting

The present study was a retrospective cohort study of eligible patients presenting to the Royal Melbourne Hospital ED over a one‐month period from 1st April 2024 to 30th April 2024. The Royal Melbourne Hospital is an adult only tertiary trauma emergency department in Melbourne, Australia. The Royal Melbourne Hospital ED cared for 88,000 patients in 2024 [9].

The study was approved by the Royal Melbourne Hospital Human Research Ethics Committee (QA 2024179).

### 2.2 Selection of Participants

We searched the Royal Melbourne Hospital's electronic medical record (EMR) system EPIC with the aim to identify all patients presenting with abdominal pain between the 1st of April 2024 and the 30th of April 2024. In practice, this involved identifying patients with the triage codes “gastrointestinal” or “genitourinary.” We then performed a manual record review and included the records of all patients over the age of 16 who were confirmed to have a primary presenting complaint of abdominal pain.

### 2.3 Data Collection

We reviewed each patient's medical record to determine their time to analgesia by identifying the patient's biological sex, the time of triage and the time the first analgesic was provided. We also recorded additional data points to assess our secondary outcomes including the patient's triage category, presenting complaint, first pain score documented, the type, dose and route of analgesia given, the patient's diagnosis at the time of discharge and their disposition. Patients who presented with abdominal pain and had no analgesia administered were included in the dataset, but were excluded from the analysis of the primary outcome (time to initial analgesia). Of note, at our hospital only medical staff and nurse practitioners are able to prescribe analgesia. Triage nursing staff are not able to initiate analgesia using nurse‐led protocols.

De‐identified data were extracted in accordance with guidance for retrospective chart review [10]. Data were collected by two researchers (LW and HP) into a pre‐determined data collection instrument in REDCap (Research Electronic Data Capture) [11]. No practice runs were undertaken before data collection began. Twenty percent of all records were reviewed by a senior researcher (EMB) to assess for accuracy and consistency. Of the records reviewed, 37 variables were identified where data were inaccurate or inconsistent. These were updated to reflect the true values. Where the interpretation of the record was ambiguous, these were flagged and resolved by consensus interpretation with a senior researcher. Data were checked by the statistical investigator (CJS) for data consistency, reasonableness and to identify outlier values. Any flagged variables were then manually checked by LW and HP to ensure accuracy prior to data analysis commencing.

### 2.4 Data Analysis

We summarised baseline characteristics using counts and percentages for categorical data, and means and standard deviations (or medians and interquartile range (IQR)) if skewed for continuous data.

For the analysis of the primary outcome, we summarised the median and IQR time to analgesia in each sex group and estimated the difference in median in time to initial analgesia using quantile regression with clustered standard errors to account for correlation within the same patient due to multiple hospital admissions.

We compared triage category by biological sex by creating a cross‐tabulation and estimated a common odds ratio using a mixed effects proportional odds model that included a random intercept. For binary and continuous secondary outcomes, we compared sex by estimating odds ratios and mean differences using mixed effects logistic or linear regression respectively. All estimates were reported with corresponding 95% confidence intervals (CIs) and *p* values.

We considered a sensitivity analysis of using multiple imputation using chained equations to handle the missing data when there was > 10% missingness in the outcome variable of interest. All analyses were conducted using Stata Version 18.0 [12]. No covariate adjustment was made given the descriptive objectives of this study.

## 3 Results

**FIGURE 1:**
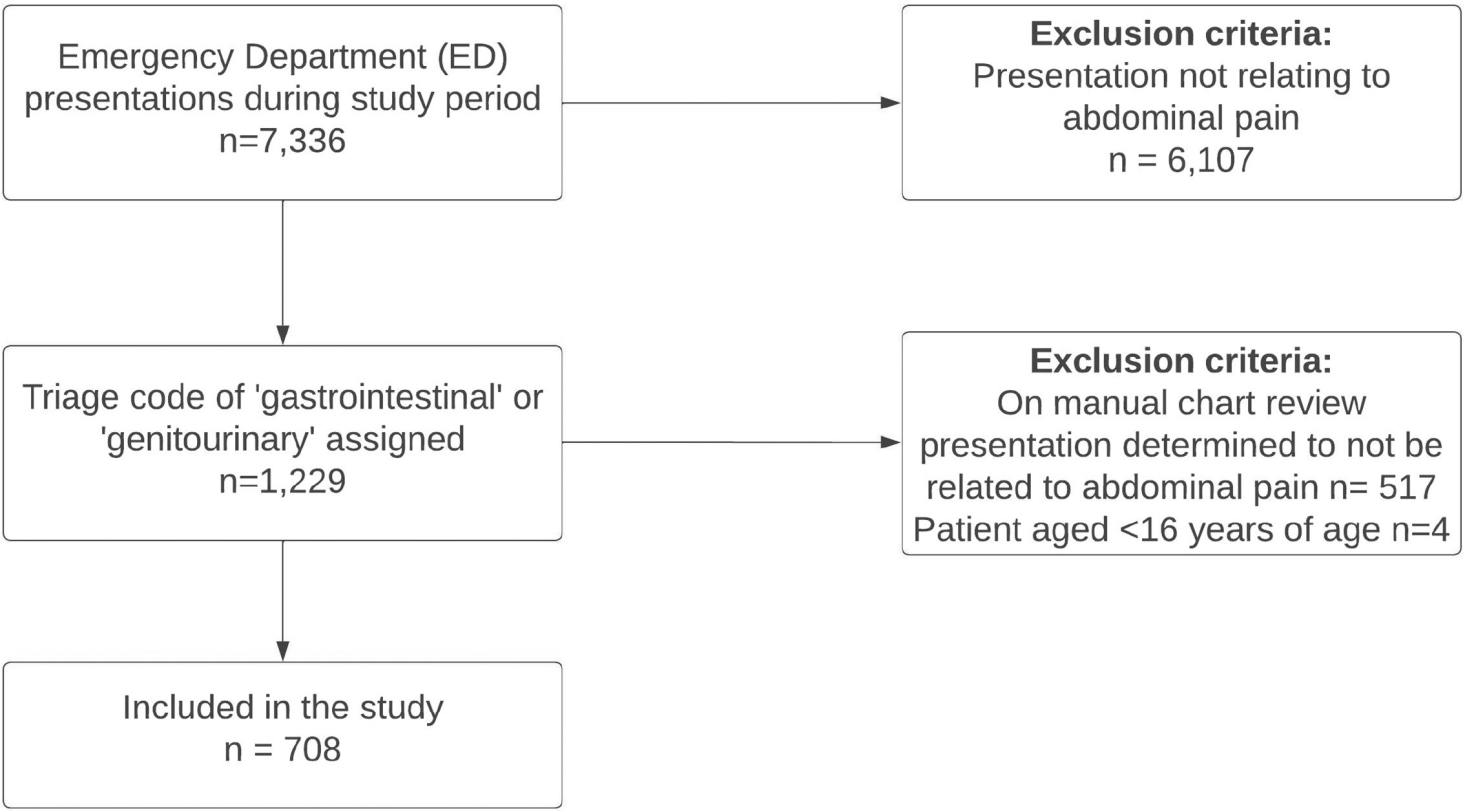
Flowchart identifying patients with abdominal pain presenting during the study period.

Over the one‐month study period during April 2024, there were 7336 patient presentations to the Royal Melbourne Hospital ED. Of these, 708 had the primary presenting complaint of abdominal pain (Figure 1). Within this patient cohort, 292/708 (41%) were biologically male and 416/708 (59%) biologically female (Table 1). The median age of the patients presenting was 40 years (Table 1). The majority 480/708 (68%) were assigned a triage category three.

**TABLE 1:** Summary of baseline characteristics of eligible patients.

	Count and percentage or mean/SD, Median/IQR
Number of eligible patients	708
Age	Median = 40 (IQR = 29.8, 57.7)
Sex	
Male	292 (41%)
Female	416 (59%)
Triage category	
1	2 (< 1%)
2	71 (10%)
3	480 (68%)
4	152 (22%)
5	3 (< 1%)

Abbreviations: IQR = interquartile range; SD = standard deviation.

### 3.1 Primary Outcome

The median time to analgesia overall for females was 75 min compared to 59 min for males. Females waited a median time of 16 min longer than males from the time of triage to receive their initial analgesia (95% CI 0.91–31.09 min, *p* = 0.04) (Table 2).

**TABLE 2:** Overall effect of biological sex on time to initial analgesia.

Females: median (IQR) time to initial analgesia (*N* = 331)	Males: median (IQR) time to initial analgesia (*n* = 228)	Median difference (95% CI)	*p*
75 min (38,140)	59 min (24, 115)	16 min (0.91 to 31.09)	0.04

Abbreviations: CI = confidence interval; IQR = interquartile range.

### 3.2 Secondary Outcomes

Around 13% of males were assigned triage categories 1 and 2 compared to only 8% of females (Table S1). Using the full triage category scale, females had lower odds of being assigned to a more urgent triage category than males, although there was considerable uncertainty in this estimate (common odds ratio 0.78, 95% CI 0.49–1.24, *p* = 0.29).

On average, males and females reported similar pain scores as measured using the numerical pain rating scale (females 5.8 [SD 2.1], males 6.0 [SD 2.4] mean difference −0.19, 95% CI −0.54 to 0.16, *p* = 0.28) (Table S2).

The proportion of females and males who received no analgesia (OR 0.91, 95% CI 0.63–1.33, *p* = 0.64) and oral analgesia (OR 1.17, 95% CI 0.79–1.73, *p* = 0.43) was similar. Females had around half the odds of receiving parenteral analgesia as men (OR 0.56, 95% CI 0.38–0.82, *p* = 0.003) (Table 3) despite the pain scores being consistent across the two populations.

**TABLE 3:** Difference in route of analgesia administration.

	Number and percentage of females across analgesia types	Number and percentage of males across analgesia types	Odds ratio (95% CI)	*p*
No analgesia	85/416 (20%)	64/292 (22%)	0.91 (0.63–1.33)	0.64
Oral	320/416 (77%)	217/292 (74%)	1.17 (0.79–1.73)	0.43
Parenteral^a^	65/416 (16%)	73/292 (25%)	0.56 (0.38–0.82)	0.003

Abbreviation: CI = confidence interval.

^a^ Due to issues with convergence with a mixed effects model, logistic regression with clustered standard errors were used to estimate the effect. An odds ratio > 1 indicates that females are more likely to receive the analgesia type.

We found minimal difference in the proportion of patients administered opioids between the biological sexes (OR 0.75, 95% CI 0.50–1.12, *p* = 0.16). There were also little differences when comparing the proportion administered opioids across the diagnostic categories assigned at ED discharge (Table 4).

**TABLE 4:** Summary of overall effect of sex on opioid use with the same diagnosis recorded at discharge.

	Number and percentage of females with opioid use	Number and percentage of males with opioid use	Odds ratio (95% CI)	*p*
Overall	160/331 (48%)	125/228 (55%)	0.75 (0.50 to 1.12)	0.16
Discharge diagnosis				
Abdominal pain acute; biliary colic/cholecystitis; appendicitis; diverticulitis; pancreatitis; hernia	103/189 (55%)	63/104 (61%)	0.68 (0.31–1.49)	0.33
Pyelonephritis/acute flank or suprapubic pain	14/33 (42%)	10/18 (56%)	0.59 (0.19–1.87)	0.37
Renal colic	9/16 (56%)	33/47 (70%)	0.49 (0.10–2.33)	0.37
Vomiting/diarrhoea; gastritis/gastro‐oesophageal reflux disease; constipation	103/189 (55%)	63/104 (61%)	0.68 (0.31–1.49)	0.33
Other	11/49 (22%)	10/34 (29%)	0.69 (0.26–1.88)	0.47

*Note:* Mixed effects logistic regression models including a random intercept for repeated hospital admissions were used to estimate the odds ratios with the exception of pyelonephritis/acute flank or suprapubic pain (where logistic regression with clustered standard errors were used due to issues with convergence). An odds ratio < 1 indicates that females were less likely to be administered opioids than males.

Disposition upon discharge was similar across the biological sexes when comparing those discharged home directly from ED, home via the ED short stay unit, and those admitted to hospital for further care (Table S3).

## 4 Discussion

This study found that patients presenting with abdominal pain who were biologically female waited a median of 16 min longer than males to receive their first dose of analgesia. We found minimal differences between sex by triage category, pain scores, or the patient's final diagnosis.

The findings of this study are consistent with previous international literature, where prospectively collected data from adults with acute abdominal pain also revealed that females were less likely to receive any analgesia and waited a mean of 16 min longer to receive analgesia when presenting to the ED for care [4, 5]. An Australian study undertaken in the private ED setting in 2023 also found that women waited 14 min longer to receive their first dose of analgesia when compared to men [8].

Males were also more likely to receive parenteral analgesia than their female counterparts. Our pre‐determined secondary analysis was to examine opioid medication use across routes rather than by specific route, but notably 86% (133/154) of parenteral medication doses provided were opioid based. Therefore, this likely represents an overall higher rate of parenteral opioid use in male patients in our cohort, a finding that is supported in the existing literature [4]. Further prospective mixed‐methods research to describe what analgesia is being provided, when and why may help to further clarify the reasons for this difference.

Disparity in pain management between the biological sexes is not isolated to the ED. A systematic review published earlier this year found that female patients' post‐operative pain was more likely to be treated with non‐opiate analgesia, and more likely to be treated with anxiolytics when compared to their male counterparts [13].

One factor that we have not explored in this paper is the role of sex concordance between the treating clinician and the patient in the provision of analgesia. In patients in the primary care setting [14] or receiving cardiology [15] and endocrinology [16] care, sex concordance does appear to affect health outcomes. For example, patients with non‐ST elevation myocardial infarctions were 5% more likely to survive if there was sex concordance between the patient and their treating physician [14]. In the ED setting, previous research has demonstrated that female patients report higher satisfaction when treated by female emergency physicians [16], but to our knowledge limited research has specifically examined the role of sex concordance on patient outcomes in the ED setting.

Future research examining the role of sex concordance and pain management should consider the role of not just the analgesia prescriber (physician) but also the analgesia provider (nurse). Due to the practicalities of how pain medication is charted for administration as a *pro re nata* medication, both the physician and nurse caring for the patient—and therefore the sex or gender of both of these parties—may influence how and when pain medication is provided.

## 5 Limitations

Our study has a number of limitations. As a single centre retrospective study, its broader generalisability may be limited. Of note, our hospital is co‐located with a tertiary women's health ED and patients with acute gynaecological presentations may therefore have been underrepresented in our study results due to the fact that patients presenting with a clear gynaecological or pregnancy related issue will be redirected to this hospital. Whilst the Royal Melbourne does see some females with gynaecological issues, this is usually those presenting with non‐specific abdominal pain, rather than those with clear pregnancy related abdominal pain and/or gynaecological pathology, who may be under represented in our data set compared to a general ED.

Additionally, due to the retrospective nature of this study we were only able to capture the time the medication was administered rather than the time the medication was prescribed. This meant we were not able to determine whether a delay to analgesia administration related to a delay in medical staff ordering analgesia or nursing staff administering the analgesia. This highlights an opportunity for further study to review the point at which the delay occurs.

Finally, we did not capture the sex of the medical and nursing staff involved in the care of these patients. Understanding the role of these sex dyads (or triads) would be a key consideration for future research.

## 6 Conclusion

This study aimed to provide further insights into biological sex disparity in the administration of analgesia to patients presenting with abdominal pain. We found that females are waiting longer than males to receive their first dose of analgesia and are less likely to receive parenteral analgesia, despite there being no clinically meaningful differences identified between diagnosis, triage categories or initial pain scores. Further research is needed to determine why these differences exist and to work towards achieving equity in analgesia provision.

## Author Contributions

L.W. and H.P. conceived the study. H.P. and E.M.B. obtained ethics and governance approval. L.W. and H.P. obtained the data. C.J.S. cleaned and analysed the data. L.W., H.P. and E.M.B. interpreted the data. L.W. drafted the initial manuscript, with all authors contributing substantially to its revision. E.M.B. takes responsibility for the paper as a whole.

## Conflicts of Interest

The authors declare no conflicts of interest.

## Supporting information


**Table S1:** Cross‐tabulation of triage category by sex.
**Table S2:** Summary of overall effect of sex on pain scores (numerical rating scale).
**Table S3:** Disposition upon discharge.

## Data Availability

The data that support the findings of this study are available on request from the corresponding author. The data are not publicly available due to privacy or ethical restrictions.
