# Supplementary material for: Time to Analgesia Provision for Abdominal Pain Presentations in the Emergency Department: The Effect of Biological Sex—A Retrospective Cohort Study
Source: Emerg Med Australas. 2026 Mar 31;38(2):e70249. doi: 10.1111/1742-6723.70249 (PMC13036702; doi:10.1111/1742-6723.70249)
Supplement: Supplementary file 1 — Table S1: Cross‐tabulation of triage category by sex. Table S2: Summary of overall effect of sex on pain scores (numerical rating scale). Table S3: Disposition upon discharge. [file EMM-38-0-s001.docx]

**Supplementary Tables**

**Supplementary Table 1:** Cross-tabulation of triage category by sex.

| **Triage Category** | **Biological Sex** | | |
| --- | --- | --- | --- |
|  | **Male** | **Female** | **Total** |
| 1 | 1 | 1 | 2 |
|  | (0.34%) | (0.24%) | (0.28%) |
| 2 | 38 | 33 | 71 |
|  | (13.01%) | (7.93%) | (10.03%) |
| 3 | 190 | 290 | 480 |
|  | (65.07%) | (69.71%) | (67.80%) |
| 4 | 62 | 90 | 152 |
|  | (21.23%) | (21.63%) | (21.47%) |
| 5 | 1 | 2 | 3 |
|  | (0.34%) | (0.48%) | (0.42%) |
| **Total** | 292 | 416 | 708 |
|  | (100%) | (100%) | (100%) |
|  | | | |

**Supplementary Table 2:** Summary of overall effect of sex on pain scores (numerical rating scale)

|  | **Females: mean (SD) pain score (N = 387)** | **Males: mean (SD) pain score (N = 275)** | **Mean difference (95% CI)** | **p-value** |
| --- | --- | --- | --- | --- |
| **Numerical rating scale - pain score** | 5.8 (2.1) | 6.0 (2.4) | -0.19 (-0.54 to 0.16) | 0.28 |

A mixed effects linear regression model was fitted to estimate the mean difference. A mean difference < 0 indicates that females reported a lower pain score on average compared to males.

**Supplementary Table 3**: Disposition upon discharge

| **Patient disposition from ED** | **Biological Sex** | | |
| --- | --- | --- | --- |
|  | **Male** | **Female** | **Total** |
| **Home direct from ED** | 81 | 120 | 201 |
|  | (28%) | (29%) | (28%) |
| **Home via ED short stay unit** | 87 | 118 | 205 |
|  | (30%) | (28%) | (29%) |
| **Admitted** | 124 | 178 | 302 |
|  | (42%) | (43%) | (43%) |
| **Total** | 292 | 416 | 708 |
|  | (100%) | (100%) | (100%) |
|  | | | |
